# From pediatrics to adult care – Experiences of transition among youth with a chronic medical condition: A meta-ethnography^☆^

**DOI:** 10.1016/j.hctj.2025.100118

**Published:** 2025-08-27

**Authors:** Bettina Trettin, Nina Hyltoft, Hanne Agerskov, Charlotte Nielsen, Christina Egmose Frandsen

**Affiliations:** a Department of Dermatology and Allergy Centre, Odense University Hospital, J.B. Winsløws Vej 2C, Odense 5000, Denmark; b Centre for Innovative Medical Technology, Odense University Hospital, Kløvervænget 19, Odense 5000, Denmark; c Department of Clinical Research, University of Southern Denmark, Health Sciences, Campusvej 55, Odense 5230, Denmark; d Department of Paediatrics and Adolescents Medicine, Herlev and Gentofte Hospital, Borgmester Ib Juuls Vej 1, Herlev 2730, Denmark; e Department of Nephrology, Odense University Hospital, Kløvervænget 6, Odense 5000, Denmark; f Department of Oral and Maxillofacial Surgery, Odense University Hospital, Kløvervænget 47, Odense 5000, Denmark; g Research Unit for Plastic Surgery, Odense University Hospital, J.B. Winsløws Vej 4, Odense 5000, Denmark

**Keywords:** Adolescents, Youth, Meta-ethnography, Qualitative, Transition

## Abstract

**Background:**

Approximately 10–30 % of the youth (aged 15–24) have a diagnosed chronic medical condition. Effective transition from pediatric to adult care is thus essential for disease management. The growing interest in the transition of young people with chronic medical conditions has led to numerous international studies revealing diverse and often inadequate transition practices. Thus, the aim was to gain a new understanding and deeper insight into youths´ experiences of their transition from paediatric to adult care.

**Methods:**

Utilizing the meta-ethnographic method by Noblit and Hare, a structured literature search

was conducted in CINAHL and PubMed.

**Results:**

Ten articles were included. The meta-ethnography found that youth – despite their chronic medical condition – define themselves as primarily young and secondarily chronically ill. Furthermore, youth transitioning to adult care are being the Captain of Their Own Life and hence stand alone with the responsibility of managing their illness, lacking the competence to master it fully, and facing an unorganized healthcare system that does not adequately support their needs. Thus, youth find they are Navigating in the Dark.

**Conclusion:**

Adopting a rigorously systematic approach to conducting a meta-ethnography provides new and valuable knowledge into the transition process from pediatric to adult care. Youth with chronic medical conditions encounter multiple challenges in their transition from pediatric to adult care, which has not systematically been integrated into patient care pathways in clinical practice. This review provides a new understanding of youths’ transition experiences, on which further research regarding the organization of effective and evidence-based process can be based.

## Highlights

• Demonstrates the need to prepare youth for transition.
• A personalized and multidimensional approach through active involvement of youth and their families may improve the experience of transition.
• Adopting a rigorously systematic approach to conducting a meta-ethnography provides new and valuable knowledge into the transition process.

## 1 Introduction

Approximately 10–30 % of youth (aged 15–24) are diagnosed with a chronic medical condition, and this prevalence may be increasing. A chronic medical condition is defined by the Centers for Disease Control and Prevention as a condition that persists one year or more and requires ongoing medical attention or has a negative impact on your daily living^1^. Effective transition from pediatric to adult care is essential for disease management and for preventing physiological and psychosocial challenges during adolescence and into adulthood^2^. Healthcare transition is the structured process of moving adolescents and young people from pediatric to adult healthcare; a process that aims to connect two entirely different systems of care. A successful transition enables youth to manage their chronic medical condition in order to become independent and take responsibility for disease management in daily life^3^, ^4^. Wisk and Sharma show an increasing prevalence of pediatric-onset chronic conditions among youth over the past two decades, highlighting the critical importance of successful transition into adult care to prevent disengagement and disease exacerbation and reduce the burden on the adult healthcare system^5^. However, these achievements appear difficult to sustain over time, even with healthcare transition support, resulting in gaps in care, poor disease management, increased morbidity and mortality, and higher rates of emergency department utilizations, and an increased need for hospital admissions^6^, ^7^, ^8^, ^9^.

Transition involves a purposeful planned approach to ensure continuity of care, enhance self-management skills and promote health outcomes^10^. For three decades, healthcare transition has been a focal point in clinical practice due to the recognition of the need to ensure a seamless transition for youth in relation to their care and health outcomes. However, despite growing awareness of transitional care, challenges related to the transition process persist^10^. A review of transition practices emphasized the need for improvements in terms of parental involvement, post-transfer support, and a consistent and structured use of transition interventions^11^. Colver et al. evaluated transition service based on nine outcome features in alignment with international recommendations. The evaluation highlighted the need to include the experiences of youth and their families, rather than relying solely on service features^8^. Gray et al. conducted a review on barriers to transition from pediatric to adult healthcare, identifying both illness-specific and general chronic illness-related barriers^12^. Theoretical frameworks or transition models were only sparsely employed, with no alignment in the chosen approach. Further, only limited evidence supported the effectiveness of transition interventions^12^.

The investigation of transitional care for youth with chronic medical conditions reveal diverse and often inadequate transition practices from pediatric to adult care. There is a need for structured transitional care, yet it must also be flexible, individualized, and involve a family perspective. Chronic medical conditions encompass a variety of diseases, with asthma and type 1 diabetes being among the most common in children and young people^13^, and cerebral palsy being the most prevalent childhood disability^14^. Health care transitions related to these chronic medical conditions, have been previously explored in the literature however limited to single studies. Conducting a meta-ethnography of youths’ experiences transitioning from pediatric to adult care across three different chronic medical conditions would allow for the synthesis of qualitative insights that transcend condition-specific contexts. Integrating findings across these three medical conditions highlight the shared experiences in care. Thus, this review can reveal structural and relational patterns within health care that either hinder or facilitate meaningful transition, thus inform clinical practice.

Furthermore, these chronic medical conditions significantly affect individuals’ everyday lives, and a successful transition is crucial for positive health behaviours, independent self-management skills, and improved health outcomes that remain crucial into adulthood^6^, ^12^. Therefore, the review focuses on asthma, type 1 diabetes and cerebral palsy with the aim to gain new understanding and deeper insight into youths’ experiences of their transition from pediatric to adult care. Furthermore, it is the intention to present a rigorously systematic approach on how to conduct a meta-ethnography.

## 2 Methods

**Fig. 1:**
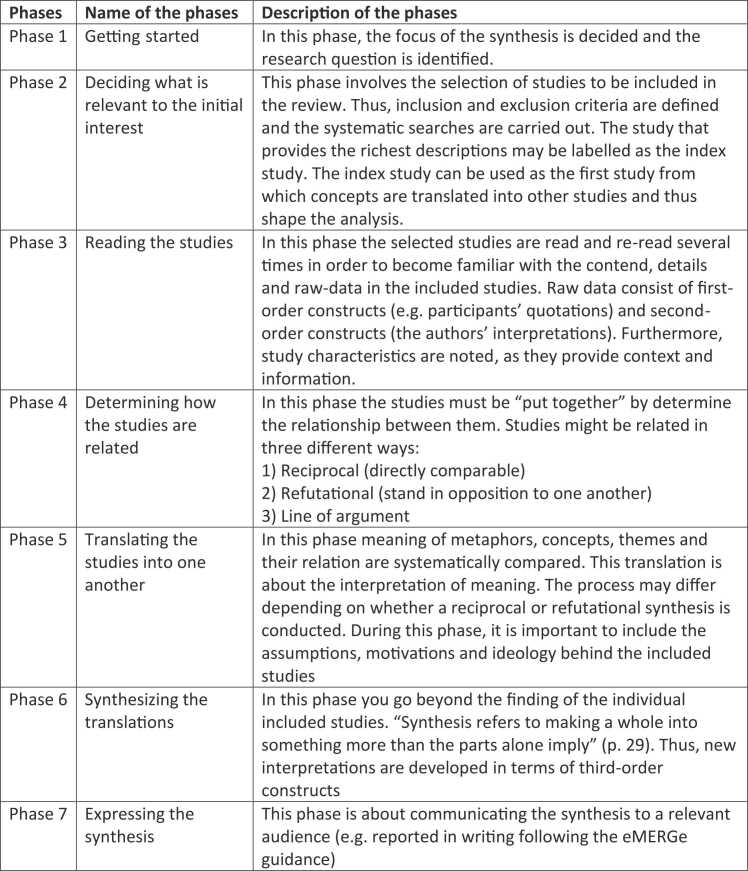
Description of the seven phases in a meta-ethnography by Noblit and Hare^17^.

Because the aim of the review was to gain new understanding and deeper insight into youths’ experiences of their transition across chronic medical conditions, conducting a meta-ethnography was a suitable choice. Meta-ethnography is the most commonly utilised qualitative synthesis approach and enables re interpretation of qualitative research results and can lead to new knowledge and theoretical perspectives^15^. Furthermore, meta-ethnography reviews present greater description of methods and higher order interpretation compared to other narrative literature reviews^16^. Noblit and Hare^17^ originally developed this interpretative method of synthesizing qualitative research and named it a meta-ethnography. Their meta-ethnography approach, which consists of seven phases, was adapted in the present review (Fig. 1). There has been criticism of the reporting of meta-ethnographies and it is acknowledged that the analytical phases (especially phase 4–6) are complex to articulate^18^. Thus, the reporting of this review followed the eMERGe guidance to enhance the transparency of the process and thus the findings^19^, which is phase seven of the meta-ethnography^15^^.^

### 2.1 Phase 1: Getting started

The meta-ethnography was registered at Prospero with ID: CRD42023476066 with the following research question: What are the experiences and perspectives of youth with a chronic disease regarding their transition from pediatric to adult care?

### 2.2 Phase 2: Decide what is relevant to the initial interest

#### 2.2.1 Selection Criteria

The inclusion criteria comprised qualitative original research studies investigating the experiences and perspectives of youth with asthma, type 1 diabetes, and cerebral palsy regarding their transition from pediatric to adult care. No limitation were set for year of publication in order to include all relevant studies. Exclusion criteria included other somatic and chronic diseases and psychiatric diseases. Furthermore, studies not written in English or a Scandinavian language were excluded to prevent cultural and linguistic bias in translation. In addition, non-peer-reviewed studies were excluded to ensure the scientific level of the studies.

#### 2.2.2 Literature search

Preliminary searches were conducted in CINAHL, PubMed, and Google Scholar. Furthermore, national and international guidelines and studies concerning transition from pediatric to adult care were retrieved. In addition, relevant grey literature such as masters and PhD dissertations were reviewed. The purpose of the preliminary searches was to identify experiences and challenges with transition among youth. Furthermore, the searches helped identify relevant search terms, thereby strengthening the systematic searches. For example, the age defining ‘youth’ varied across the retrieved studies. For this reason, a specific age range was not defined in the inclusion and exclusion criteria.

**Fig. 2:**
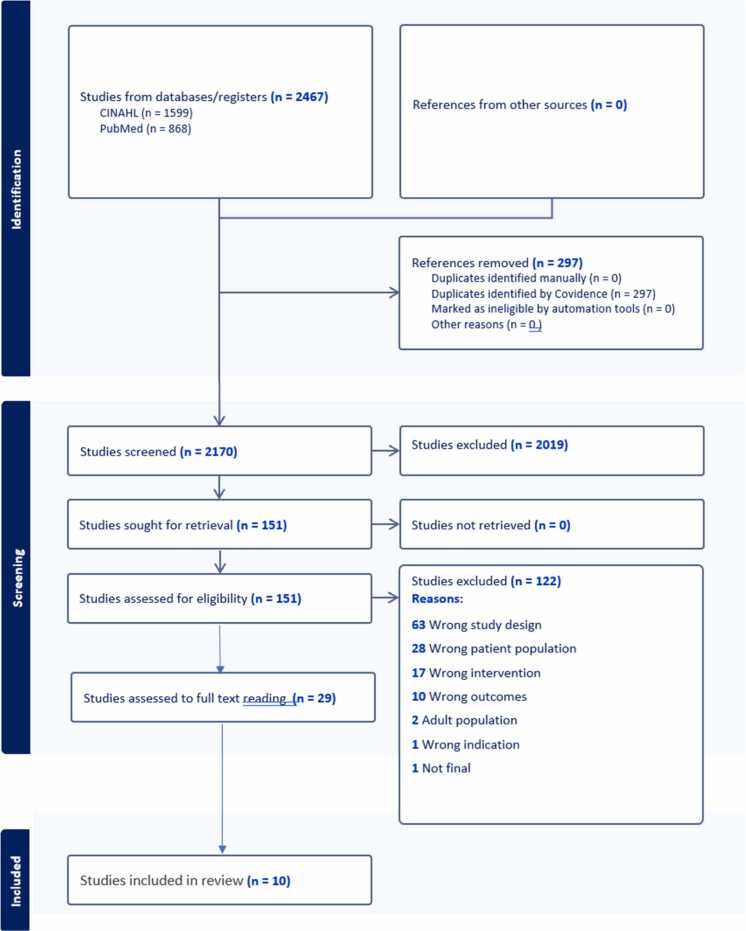
Flowchart.

The systematic literature searches were performed in the PubMed and CINAHL databases using a detailed search strategy (Supplementary file 1 +2). The search was conducted in October 2023 in close collaboration with a university librarian. The search terms used are presented in Table 1. A hand search was also performed of the reference lists of the included studies. The Covidence software was used as a reference management tool. One author (NH) screened the records for inclusion and two authors (BT and CEF) checked the decisions. Any uncertainty or disagreements were resolved by involving the entire research group. In total, ten studies were included in the meta-ethnography (Fig. 2 flowchart).

**Table 1:** Search terms used in the systematic literature search.

	Population BLOCK 1	Exposure BLOCK 2	Outcome BLOCK 3
OR	Child Children Adolescent Adolescents	Chronic disease Asthma Type 1 diabetes Cerebral palsy	Transition to adult care Transitional care Transfer Transition
< -AND ->

#### 2.2.3 Quality appraisal

To assess the relevance and credibility of the included studies, each primary study was appraised using the Critical Appraisal Skills Programme (CASP) criteria for evaluating qualitative research^20^. Noblit and Hare^17^ encourage a pragmatic approach in the appraisal of included studies. Thus, studies were evaluated to appraise the degree to which they provided a rich account of youths’ experiences of their transition and not solely the studies’ quality of the reporting. Based on Noblit and Hare, the study with the highest score (providing the richest descriptions) was designated as the ‘index’ study and served as the foundation from which concepts were translated into the other studies^16^, ^21^.

### 2.3 Phase 3: Reading the studies (Data abstraction)

All included studies were read several times to familiarize ourselves with the key concepts and metaphors. During this process, study characteristics for each study were extracted because it provided context (sample, data collection methods, data analysis, study outcome, study conclusion) for the interpretation and explanation of each study (Table 2)^16^. Simultaneously, first-order and second-order constructs were extracted using a standardized data extraction form (Table 3).

**Table 2:** Characteristics of included studies.

**Characteristics of included studies**						
Author and year	Study objective	Study population	Methods and study design	Major themes	Study conclusion	CASP score
1. McLaughlin 2013, USA^22^	Uncovering young people's experiences regarding the transition from pediatric to adult care to identify a central structure relevant to nursing care for young people with cerebral palsy	Age range 18–25 Female, 6 Male, 3 Diagnosis: cerebral palsy	Exploratory design Inductive phenomenological analysis Unstructured open interviews	Expert novices Negotiating new systems Interdependence Accepting less than was expected	More information and support are needed during transition Nurses’ role as advocate, mentor and guide can optimize the individual’s response to the transition process.	9
2. Strand 2018, Norge^23^	How young people experience the transition from being dependent on their parents to managing their own type 1 diabetes, with a focus on improving diabetes nursing care in both pediatric and adult settings	Age range 16–18 Female, 11 Male, 7 Diagnosis: type 1 diabetes	Exploratory design Inductive phenomenological analysis Individual interviews	Taking responsibility for own diabetes is a process Taking responsibility for own diabetes was dependent on coping It is demanding to take responsibility for own diabetes	Adolescents want to take over the responsibility for their diabetes treatment but need knowledge, experience and skills to succeed Parents, friends and health professionals are important supporters during the transition	8
3. Larivière-Bastien, 2013, Canada^24^	Exploring whether and how young people feel respected during the transition process: Integration of their values and preferences, and preparation for decision-making autonomy for young people with cerebral palsy	Age range 18–23 Female, 7 Male, 7 Diagnosis: cerebral palsy	Semi-structured interviews, Preceded by a questionnaire. Thematic content analysis	Transition envisaged with fear and apprehension Lack of cooperation or communication between providers in the pediatric and adult healthcare systems Lack of support, preparation, and information Difficulties related to the differences between the two healthcare systems Abrupt loss of services; sensing a void at the time of transition Feelings of abandonment during the transition	More substantial recognition of the importance of the values of individuals with CP and respect for them as persons Improved preparation for autonomous decision-making	7
4. Björquist, 2014, SE^25^	Gaining a deeper understanding of how young people with cerebral palsy experience their health, well-being, and support needs during their transition to adulthood: Improving the organization of the transition process by uncovering their experiences	Age range: 17–18 Participants: 12 Diagnosis: cerebral palsy	Individual and focus group interviews using an interview guide and illustrations with pictures and pictograms. Manifest and latent analysis	Belonging to a family The importance of friends and love Managing daily activities Being surrounded by support Having hopes for the future	The support should be flexible and not be fixed to biological age Desire for personal, individualized information and support	6
5. Leung, 2020, Canada^26^	Exploring young people’s perspectives and experiences to design and implement future transition interventions, improve health and patient satisfaction for young people with type 1 diabetes at a Canadian hospital, and implement future transition interventions	Age range: 16–18 Female: 13 Male: 9 Diagnosis: type 1 diabetes	Focus group interviews and individual questionnaire Interpretive analysis	Individualization – how to personalize the transition experience Identity – how the world relates to my diabetes Interconnection – how my support system can help me with my diabetes Impediment – how my diabetes limits me	Transition programmes should be based on individualization, identity, interconnection, and impediment	6
6. Iversen, 2019, Norge^27^	How young people with diabetes experience the transition from pediatric to adult care	Age range 19–23 Female: 5 Male: 6 Diagnosis: type 1 diabetes	Theory grounded in phenomenology Exploratory, semi-structured interviews	Limited information about the transition Transition from frequent, thorough and personal follow-up to a less comprehensive and less personal follow-up The importance of being seen as a whole person Limited expectations of how the health care services were organized	Existing routines for transfer are not optimal Participants expressed that they were not prepared for the dissimilarities in follow-up and were predominantly less pleased with the adult care follow-up	6
7. Castensøe-Seidenfaden 2016, DK^28^	Exploring and describing the experiences of young people with type 1 diabetes to identify their support needs and improve their ability to self-manage during the transition from childhood to adolescence	Age range: 15–19 Female: 5 Male: 4 Diagnosis: type 1 diabetes	Individual interviews with photos Thematic content analysis	Striving for safety, Striving for normality Striving for independence Worrying about future	The concerns and challenges adolescents and their parents face during the transition from childhood to adulthood are still present, despite new treatment modalities Parents are fundamental in supporting the adolescents’ self-management-work; however, the parties have unspoken concerns and challenges	5
8. Olsson, 2023 SE^29^	Exploring young adults’ experiences in the transition to adulthood, including their experiences with type 1 diabetes in the transfer from pediatric to adult care: Describing the variation in transition experiences regarding type 1 diabetes	Age range: 19–29 Female: 6 Male: 4 Diagnosis: type 1 diabetes	Semi-structured and open interviews Qualitative content analysis with manifest and latent analysis	Struggling to find balance in daily life Dealing with feelings of being different Being gradually supported to achieve independence, and wishing to be approached as a unique person in healthcare	Important to emphasize not only diabetes-related factors but also emotional and psychosocial aspects of life connected to the transition to adulthood, including the transfer to adult care	4
9. Rhee, 2022, USA^30^	Investigating young people’s experiences and insights with asthma, parental involvement in asthma care, and communication with healthcare professionals regarding the transition to adult care	Age range: 16–20 Female: 17 Male: 24 Diagnosis: asthma	Focus groups and individual interviews Content analysis	Concerns about transitioning into adulthood with asthma and managing the condition independently Parental involvement Communication with providers	A partnership between parent, provider, and adolescent is essential for fostering adolescent readiness for independent asthma management and ensuring continuity of care after the transition	4
10. Price, 2011 UK^31^	Evaluating a transition pathway and understanding the unique experiences of young people with type 1 diabetes after completing the transition process through four check-ups	Age range: 16–18 Participants: 4 Diagnosis type 1 diabetes	Semi-structured interviews Interpretive analysis	Transition services need to be developmentally appropriate and based on individual needs	Professionals need to understand adolescence as a ‘life stage’ with all its biological and psychosocial changes, as well as communicate effectively with young people on an individual basis when engaging with them as patients	2

**Table 3:** Example of first-order and second-order constructs and the primary author’s concepts.

Study title: Perspectives of young adults with cerebral palsy on transitioning from pediatric to adult healthcare systems Objective: investigate the experiences of youth prior, during, and after transition and whether they felt respected during the transition. Findings: The transition identified eight distinct tension points captured as a process		
**From constructs to concepts**	**Participant quote #1**	**Participant quote #2**
First-order constructs	*‘…But, you know, really, even if you’re older than 18, the disability is still there.(…) the 18-year-mark isn’t magic, you know! (Laugh) We still have a lot of needs, you know…’* *“…A lot of people are happy to turn 18, but myself…I didn’t like it! (Laugh) I found it hard…. Really? You thought I wasn’t an adult at 17, but now that I’m 18, I become one and I have to listen to what you have to say. OK, this* *is a little bit strange…’*	*…[I] am a little bit, uh,* *disoriented, but a little bit sad* *too, for having left. Because,* *without going into the details,* *it’s like a family when you* *grow up, for 21 years, with a* *family, well, [name of a* *pediatric hospital] or [name of* *another pediatric hospital] or* *[name of another pediatric* *hospital], you grow up with* *them. We saw each other and,* *‘Hey, it’s [name of the* *participant]!!’ So the fact of* *leaving all this, it’s like leaving* *a part of my family, so it’s* *hard…’*
Second-order constructs	It was the abruptness of the transition that participants found most disruptive. They often expressed a sense of losing everything overnight and were unable to access the same services in the adult system. They pointed out the absurdity of feeling like a radical change was expected when they turned 18 years old.	Another common feeling among the participants who have gone through the transition is the sadness of having left the pediatric system, its services, and above all, the relationships they have developed there.
Concepts	Abrupt loss of services, sensing a void at the time of transition	Emotional impact of leaving the pediatric system

### 2.4 Phase 4: Determining how the studies are related

In this phase, the relationships between the key concepts from the included studies were considered in order to determine how they were related. This was done by systematically and structurally assessing how the key concepts from each study were related, while also acknowledging that this process remained inductive and flexible. The five key concepts from the ‘Index’ study (Table 4) were successively compared to the key concepts of the included studies one at a time (Key concepts from all included studies available in Supplementary fil 3). This was accomplished by creating a list of concepts from each study. The concepts were listed under the name of each study, and both first-order and second-order constructs were placed in their respective columns. After testing various flexible methods, an analogue approach was chosen. This analytical phase of the meta-ethnography involved cutting out the concepts on paper and creating an overview of the main concepts across studies, based on the index study’s core concepts. It was an extensive and iterative process carried out over several weeks. A third column was used to note the contexts, goals and focus of the studies to maintain an interpretation that included these perspectives because they were considered valuable^17^. This process formed the basis for subsequently presenting the main concepts in a translation matrix, illustrating how the studies were related to one another (Supplementary file 4). In this phase, we determined that a reciprocal translation was suitable, as the concepts from the index study were comparable and could encompass those from the other studies. This was followed by a line-of-argument synthesis, which was considered the next step in the analysis^16^. The line-of-argument synthesis involves translating diverse interpretations of different aspects of the same phenomenon and integrating them into a coherent whole that surpasses the mere sum of its individual components.

**Table 4:** Key concepts from the index study.

**Key concepts from the index study**
Expert novices
Evidence and experience-based expectations
Negotiating new systems
Interdependence
Accepting less

### 2.5 Phase 5: Translating the studies into one another

In this phase, the reciprocal translation of the studies was performed. According to Noblit and Hare, it is crucial to evaluate the studies with respect and to safeguard the unique findings, particularly the experiences that emerge within the individual qualitative studies. Appropriate translation thus requires preserving the distinct meaning of each study while systematically comparing key metaphors and concepts across studies, while integrating them by analyzing their relationships and interactions^17^. Thus, phase 5 is reported in a translation table (Table 5) and a narrative example of how the key concept “expert novice” was translated (Table 6). The translation table provides an overview of how the key concepts from the included studies are distributed and thus, creates visibility and overview of the structure and interrelationship of the included studies. During this process, residual concepts were identified. Residual concepts were concepts that did not fit into the ongoing analysis but still revolved around youths’ experiences. Next residual concepts from the 10 studies were assessed iteratively to find common characteristics. This led to the development of one additional key concept that did not originate from the index study but was considered to contribute with valuable insights and experiences according to the aim of this meta-ethnography; see Translation Table 5.

**Table 5:** Translation table.

**Key concepts**	**McLaughlin 2013**	**Strand** 2018	**Larivière-** **Bastien** 2013	**Björquist** 2014	**Leung** 2020	**Iversen** 2019	**Castensøe** **Seidenfaden** 2016	**Olsson** 2023	**Rhee** 2022	**Price** **2011**	**Total**
Expert novices	*				*	*	*		*		5/10
Evidence and experience based expectations	*		*		*			*	*	*	6/10
Negotiating new systems	*	*	*	*	*	*				*	7/10
Interdependence	*			*	*		*	*	*		6/10
Accepting less	*		*			*			*	*	5/10
**Key concept from categorization of residual concepts**											
Being a person with a disease		*		*		*	*			*	5/10

**Table 6:** Examples of the narrative translation of the studies based on the key concept *expert novice*.

**Expert Novices**					
Interpretations of the studies and their relations to the index study					
**Mclaughlin 2013** **(index study)**	**Leung, 2020**	**Castensøe, 2016**	**Iversen, 2019**	**Rhee, 2022**	**Towards third-order constructs**
Young people aged 19–25 retrospectively view themselves as experts in their own illness and care, while simultaneously felt as novices. After transitioning to adulthood, they experience new issues related to living with a chronic illness and want to discuss these issues with professionals in adult care settings.	Young people experience stigmatization and discrimination at both micro and macro levels due to their chronic illness. Young individuals find their chronic illness annoying in social interactions with friends and report feelings of social exclusion in societal contexts. Despite having lived with their illness and been supported by knowledgeable professionals for an average of nine years, they are faced tackling these new challenges as perceived novices.	Chronically ill young people aged 15–19 strive for a sense of normalcy, seeking a youth experience free from illness and the need for to take special accommodations in their daily lives. They perceive themselves as experts in their own illness and understand the consequences of non-compliance. Nevertheless, they choose to skip planned medical check-ups to hide their lack of knowledge or ability to comply, which constitutes the novice part and leads to under-treatment. The lack of professional support hinders the autonomy of the young individuals. They know what is required in relation to their chronic illness but fail to integrate it into their new life as independent young individuals, hence the novice label.	Young people are unprepared for the transition to adult care, despite being diagnosed with their chronic illness for 9–19 years. These young individuals call for oral and written information well in advance of the transition. Some young people with chronic illness desire preparation nine months in advance and prefer oral information. Others seek professional information about the transition throughout the patient journey from both pediatric and adult care settings.	Despite choosing to underuse their medication, chronically ill young people maintain a high level of self-confidence. This is identified with the perception of being an expert in their own illness and actively choosing to avoid asthma medication. This leads to under-treatment with the risk of long-term health consequences, hence the novice label. Rhee also finds that chronically ill young people who have lived with their illness for at least a year prioritize a youth experience free from medication	Based on first-order and second-order constructions, it is assessed that young people across nationalities and diagnoses perceive themselves as experts in their own illness, while simultaneously being novices in navigating adolescence with a chronic illness. The experience of transition is independent of the duration of contact with health professionals in pediatrics. Encounters with adult care professionals lead to uncertainty and are perceived as intimidating, as the professionals do not provide clear information and knowledge, leaving these matters up to the young individuals. Thus, they experience being expert-novices.

**Fig. 3:**
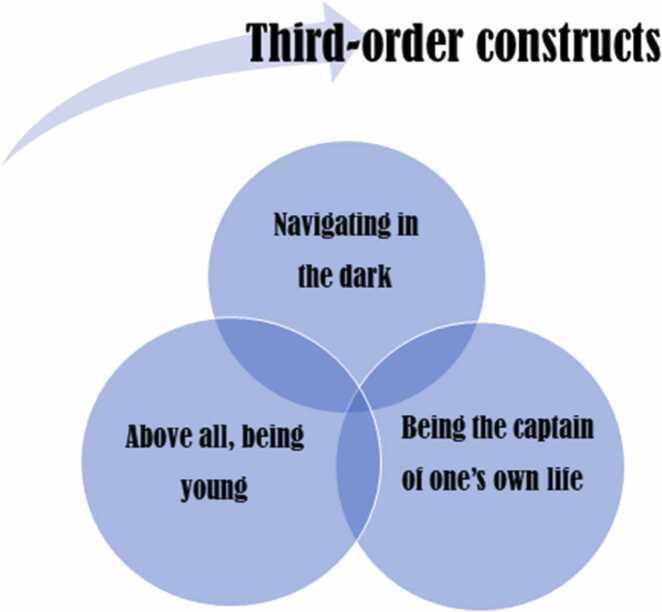
Third-order constructs.

The key concept “expert novice” is presented through a narrative account of youths´ experiences with the transition from paediatric to adult care, analysed with attention to the distinct characteristics of each individual study. In order to foster a transparent reporting of how third-order constructs emerged, an example of how the key concept “expert novice” was analysed is presented in Table 6. The translations of the studies, therefore, incorporated both first-order and second-order constructs as justification for the interpretations. All key concepts presented in the translational Table 5 were analysed as shown in Table 6. The column in Table 6: Towards third-order constructs, are new interpretations that emerged from the included studies and thus represent new findings across studies. These new interpretations (findings) were used to generate the third order constructs presented in Fig. 3.

## 3 Phase 6: synthesizing the translations

In this phase, you go beyond the findings of the individual included studies and create a new whole (Fig. 1). New interpretations emerged in terms of third-order constructs. The third-order constructs serve as recurring metaphors and thus foster a deeper understanding of the experiences and perceptions of youth with chronic medical conditions and their transition. The third-order constructs are presented in Fig. 3.

### 3.1 Navigating in the dark

Upon completing either short-term or long-term pediatric care, youth with chronic medical conditions felt like they were navigating in the dark. They experienced the transition as an ending that brought about a sense of loss and the severing of close relationships with healthcare professionals. This sense of loss was consistent across nationalities and regardless of the type of chronic medical condition, stemming from their experience-based expectations in pediatric care^22^, ^24^, ^29^. Youth felt unprepared for the transfer and believed that their time in pediatric care had not adequately prepared them for the transition or for living with a chronic illness as a young person. They also felt that healthcare professionals in adult care did not support their need to learn how to live with a chronic illness^22^, ^24^, ^26^, ^27^, ^28^, ^29^, ^30^, ^31^. During the transition, they found themselves being unstructured and unprepared, without guidance, and risking health consequences. They felt like ‘expert novices’, having to manage new activities on their own. They encountered exclusionary and negative social stereotypes that challenged their identity development during adolescence^26^, ^31^. They felt as though they were navigating in the dark, lacking the necessary experience or guidance to manage their illness^24^, ^26^, ^29^, ^30^, ^31^.

Youth with chronic medical conditions also felt abandoned when they lost their compass in the form of relationships with pediatric healthcare professionals and at the same time experienced changes in their parental relationships^22^, ^25^, ^26^, ^28^, ^29^, ^30^. Some also felt as though they were navigating in the dark for other reasons: they found that youth activities were incompatible with impulsive and logistical activities, and they felt unprepared and uncertain when it came to forming friendships and romantic relationships, which were important elements in their lives^22^, ^25^.

### 3.2 Being the captain of one’s own life

The experience of being the captain of your own life was closely linked to the previously mentioned third-order construct of navigating in the dark. Youth with chronic medical conditions experienced that they were given full responsibility for their lives during the transition from pediatric to adult care^22^, ^23^, ^24^, ^25^, ^26^, ^27^, ^28^, ^29^, ^30^, ^31^. Despite long-term support from healthcare professionals in pediatrics, they felt abandoned during the transition, lacking the expertise to manage their illness and without proper communication between sectors, such as medical records or physiotherapy needs^24^, ^25^.

They generally felt that the healthcare system fell short of their expectations, casting them as captains of their own lives, but without the necessary skills to steer the course. This lack of competence and support from professionals was disappointing and made managing their condition a burdensome responsibility. Some felt fully able to manage their condition, while others felt overwhelmed. The common experience was feeling alone in bearing the responsibility for managing their condition^22^, ^23^, ^25^, ^26^, ^30^.

The fear of making treatment mistakes added pressure, akin to being examined^22^, ^23^, ^24^. This duality (possessing both responsibility and knowledge of their illness, yet not seeking support despite needing it) intensified their sense of inadequacy.

### 3.3 Above all, being young

Youth with chronic medical conditions saw themselves as young individuals first, and as chronically ill second. During the transition from pediatric to adult care, they felt their youthfulness dominated their experience, and their chronic illness was more than just an age-specific issue^23^, ^24^, ^25^, ^26^, ^27^, ^29^, ^30^, ^31^.

Youth expected a transition process where healthcare professionals engaged with them both personally and professionally. They felt that adult care did not offer the personal relationships they had built up with pediatric professionals. They desired a personalized, multidimensional approach rather than the standardized one offered by adult care^26^, ^27^, ^30^. In adult care, they experienced a lack of time and resources, leading to a sense of loss and sadness over leaving the familiar pediatric environment. They preferred connections with mentors - or peers with chronic medical conditions - to support them during transition^24^, ^25^, ^26^, ^30^.

Managing their illness at a suitable pace was important for their sense of achievement. They valued interventions that supported self-efficacy and empowerment, which helped them accept their condition^29^. Socializing and experiencing love were challenging due to their condition, and they felt unprepared to form friendships and romantic relationships^22^, ^25^, ^29^. Stigmatization in public due to negative stereotypes about chronic medical conditions negatively impacted their quality of life, making it difficult to feel in control of their lives^26^.

## 4 Discussion

This review aimed to gain a new understanding and deeper insight into youths’ experiences of their transition from pediatric to adult care when living with a chronic medical condition. The analysis showed a range of experiences during transition. First, youth desired to be seen as a person, with the disease being secondary. Next, feeling safe in clinical settings and in relationships with healthcare professionals was considered significant when transitioning from pediatrics to adult care. Feelings of not being seen, heard, or met provided experiences of being alone and navigating in the dark.

A study by Fortune et al.^32^ showed that youth found that a lack of information, preparations and continuity prevented successful transition. Further, the authors found that the complexity of the transitional process means that youth need a holistic supportive approach and a system which includes all relevant stakeholders –interactions in the family, between HCPs and between sectors^32^. In our review, we found that the youth with chronic medical conditions primarily wanted to be seen as a young person and that their illness was secondary (Above all, being young) to their person and identity. Cass et al.^33^ report that the way youth are met and approached is of great importance, and they recommend and support the importance of a holistic approach. Transitioning from pediatrics to adult care is a period of extensive change on many levels^32^. In the present review, the youth found it akin to ‘navigate in the dark’, which included feelings of loss, the severing of close relationships with healthcare professionals, and being unprepared for the transfer into adult care.

Peeters et al.^11^ emphasize the importance of focusing beyond the transfer period by providing early guidance and education to youth. Continued contact with the healthcare system should also be prioritized to prevent acute hospitalizations and non-adherence to treatment. Further, the authors describe the important role of the parents, stressing the need to involve and educate them on how to support the young person in their new role as an adult. The present review showed that the youth during the transition to adult care also experienced changes in their parental relationships, towards being captain of their own life.

According to Meleis^34^, transition is a process of three phases with a beginning, a passage, and an end. Transition is part of human life and can arise as a result of external factors. It may occur at different paces and can involve one or more people^34^. Transitioning is related to 1) Maturation: the developmental phase that adolescent life entails; 2) Illness: the chronic illness; 3) Experiences of loss: when contact to known healthcare professionals ends. A failed transition can according to Meleis’, lead to emotional distress, insecurity and disruption in roles and relationships.

With this theory in mind, youths’ insecurity varies upon completing either short-term or long-term pediatric care, hence, they feel like navigating in the dark. Thus, Meleis’ transition theory can explain the need for an individualized approach, as transition is a process that unfolds over time and, the impact of transitional events varies between individuals

In the present review it was evident that healthcare professionals in pediatric care were able to tailor their approach to each young person. However, in adult care youth experienced daily life and interactions with healthcare professionals as unpredictable. They felt they were not met on an equal footing, resulting in a sense of unpreparedness for transition and thus being alone as the captain of their own life. Additionally, they felt reliant on their families, but also found that their families could be overwhelming. Peeters et al.^11^ described the role of parents as valuable, involving an ongoing partnership across pediatrics and adult care. In their study, they highlight the importance of person-centred nursing consultation for adolescents and their parents to facilitate coping of new mutual roles^11^.

With a focus on the family as a unit, the purpose is to identify family members’ perceptions of a current situation and how relationships between them are affected by life circumstances such as chronic medical conditions. When delivered in a supportive way, family nursing interventions have proved effective in improving family functioning^35^, ^36^. The present review focused on youth with chronic medical conditions’ experiences during transition; a period where parents must reflect on how to withdraw their need for control and responsibility for their child’s illness. Furthermore, siblings are an integrated part of the family and are affected by changes in the family^37^, ^38^.The review showed that ‘Above all, being young’ reflected how youth with chronic medical conditions were challenged in their identity development and felt they did not possess the necessary experience or training to manage their condition. Thus, all family members are in transition and may need support to manage daily life to keep the family functioning. According to Wright and Leahey^39^, family conversations serve as a means to engaging with families to provide guidance and support. The family conversations should be balanced, non-hierarchical, and include all family members. Questions should be open, explorative and circular in nature, encouraging reflection among family members^40^. In the present review, youth with chronic medical conditions expressed a desire to be first and foremost perceived as young individuals, without experiencing an abrupt sense of loss during the transition. Even though they appreciated interventions that promoted self-efficacy and empowerment, it is questionable whether youth fully grasp the significance of the transition process. When discussing the findings of this review, it becomes evident that to fully support youth with chronic medical conditions during the transition process from pediatric to adult care, a holistic approach is important – and should focus on both their individual needs and the functioning of their families.

### 4.1 Strengths and limitations

A significant strength of this review was employing the meta-ethnography as a method to synthezise qualitative research about youths’ experiences with transitioning from pediatric to adult care. The methodology provided an opportunity to share the outlook of youth with different chronic medical conditions. A limitation of this review was that literature searches were conducted using only two databases. Utilizing more databases could have incorporated additional literature into this review. However, 10 comprehensive studies were found, which brought rich data into this meta-ehnography in order to gain insights and a deeper understanding of the experiences among youth with chronic medical conditions regarding their transition from pediatric to adult care. All the included studies were from Western countries, which is a strength to the tranferability of the findings into Western clinical settings. Furthermore, it is considered a strength that the synthesis was reported with examples on the movement from first-order and second-order order constructs to third-order contructs to ensure the transparancy of the review.

Including primary studies addressing different chronic medical conditions may be a limitation in this review. While cerebral palsy may exert a more profound impact on youths’ daily lives and thereby place greater demand on healthcare professionals during transition, conditions such as type 1 diabetes and asthma, may pose quite different challenges. The synthesis may also be shaped by the disproportionate representation of studies involving young people with type 1 diabetes and the inclusion of one single study on asthma. These imbalances potentially affect the weighting of the findings. However, meta-ethnography is considered an appropriate methodology, as the phenomenon of 'transition' encompasses complex experiences, and the approach has enabled an exploration of the transition process from multiple perspectives and within diverse contexts. It is acknowledged that the findings of a meta-ethnography generate interpretative insights rather than definitive knowledge claims. Furthermore, the meta-ethnography has been carried out systematically and adheres to the requirement for transparent reporting of all stages, which has been iterative, reflective, and comprehensive.

### 4.2 Implications for clinical practice and research

This meta-ethnography may serve as a foundation for structuring and targeting clinical practice efforts aimed at improving the current transition process. It is essential that the transition is facilitated by the appropriate healthcare professionals to ensure continuity of care across paediatric and adult services. Furthermore, it may have implications for transition practices by encouraging professionals to plan individualized and personally tailored care pathways, and to engage with each young person primarily as a young individual—within a present and person-centred relationship. Based on this review appropriate transistion interventions across paediatric and adult care settings are needed, which therefore warrants further investigation to design a comprehensive healthcare solution for youth, their families and clinical practice.

## 5 Conclusion

In conclusion, adopting a rigorously systematic approach to conducting a meta-ethnography provides new and valuable knowledge into the transition process from pediatric to adult care.

The transition has a major impact on youths’ exeperience of the care provided to them, regardless of the underlying chronic medical condition. The experience of feeling abandoned without the necessary skills to cope with the condition contrasts with the desire to simply be young. Thus, there is significant potential to improve the transition process through a more personalized and multidimensional approach. This entails structured collaboration and active involvement of the youth and their families, facilitated by healtcare professionals.

## Funding statement

The authors did not receive any funding for this work.

## Credit statement

NH, CE and BT were involved in the study conceptualization, data curation and analysis and in the writing of this manuscript. CN and HA were involved in the writing of the manuscript.

## Funding

This research did not receive any specific grant from funding agencies in the public, commercial, or not-for-profit sectors.

## Ethical statement

Data in this manuscript was extracted from published studies, thus, no ethical approvals were necessary.

## CRediT authorship contribution statement

**Christina Egmose Frandsen:** Writing – original draft, Supervision, Methodology, Investigation, Formal analysis, Data curation, Conceptualization. **Charlotte Nielsen:** Writing – original draft, Supervision. **Hanne Agerskov:** Writing – original draft, Supervision. **Nina Hyltoft:** Visualization, Investigation, Formal analysis, Data curation, Conceptualization. **Bettina Trettin:** Writing – original draft, Visualization, Supervision, Methodology, Investigation, Formal analysis, Data curation, Conceptualization.

## Declaration of Competing Interest

The authors have no competing interest to declare.

## Data Availability

No data was used for the research described in the article.
