# Supplementary material for: From pediatrics to adult care – Experiences of transition among youth with a chronic medical condition: A meta-ethnography
Source: Health Care Transit. 2025 Aug 27;3:100118. doi: 10.1016/j.hctj.2025.100118 (PMC12408251; doi:10.1016/j.hctj.2025.100118)
Supplement: Supplementary file 1 — Supplementary material [file mmc1.pdf]

An official website of the United States government
Here's how you know

PubMed Advanced Search Builder

PubMed®

Filters applied: Danish, English, Norwegian, Swedish. Clear all

Add terms to the query box

All Fields

Enter a search term

ADD

Show Index

Query box

Enter / edit your search query here

Search

History and Search Details

Search	Actions	Details	Query	Results	Time
#14	•••		Search: **((("Transition to Adult Care"[Mesh]) OR "Transitional Care"[Mesh]) AND ((("Chronic Disease"[Mesh]) OR "Diabetes Mellitus, Type 1"[Mesh]) OR "Cerebral Palsy"[Mesh])) OR (((child OR children OR adolescent) AND ("diabetes type 1"[title/abstract:~2] OR "cerebral palsy" OR "asthma" OR "chronic disease*")) AND (transfer OR transition) AND (2023:2023[pdat]))** Filters: **Danish, English, Norwegian, Swedish**	868	04:45:16
#13	•••		Search: **((("Transition to Adult Care"[Mesh]) OR "Transitional Care"[Mesh]) AND ((("Chronic Disease"[Mesh]) OR "Diabetes Mellitus, Type 1"[Mesh]) OR "Cerebral Palsy"[Mesh])) OR (((child OR children OR adolescent) AND ("diabetes type 1"[title/abstract:~2] OR "cerebral palsy" OR "asthma" OR "chronic disease*")) AND (transfer OR transition) AND (2023:2023[pdat]))** Filters: **Danish, English, Norwegian**	868	04:41:04
#12	•••		Search: **((("Transition to Adult Care"[Mesh]) OR "Transitional Care"[Mesh]) AND ((("Chronic Disease"[Mesh]) OR "Diabetes Mellitus, Type 1"[Mesh]) OR "Cerebral Palsy"[Mesh])) OR (((child OR children OR adolescent) AND ("diabetes type 1"[title/abstract:~2] OR "cerebral palsy" OR "asthma" OR "chronic disease*")) AND (transfer OR transition) AND (2023:2023[pdat]))** Filters: **Danish, English**	867	04:40:50
#11	•••		Search: **((("Transition to Adult Care"[Mesh]) OR "Transitional Care"[Mesh]) AND ((("Chronic Disease"[Mesh]) OR "Diabetes Mellitus, Type 1"[Mesh]) OR "Cerebral Palsy"[Mesh])) OR (((child OR children OR adolescent) AND ("diabetes type 1"[title/abstract:~2] OR "cerebral palsy" OR "asthma" OR "chronic disease*")) AND (transfer OR transition) AND (2023:2023[pdat]))** Filters: **Danish**	2	04:40:42
#10	•••		Search: **((("Transition to Adult Care"[Mesh]) OR "Transitional Care"[Mesh]) AND ((("Chronic Disease"[Mesh]) OR "Diabetes Mellitus, Type 1"[Mesh]) OR "Cerebral Palsy"[Mesh])) OR (((child OR children OR adolescent) AND ("diabetes type 1"[title/abstract:~2] OR**	905	04:40:28

Search	Actions	Details	Query	Results	Time
			**"cerebral palsy" OR "asthma" OR "chronic disease*")) AND (transfer OR transition) AND (2023:2023[pdat]))**		
#9	•••		Search: **((child OR children OR adolescent) AND ("diabetes type 1"[title/abstract:~2] OR "cerebral palsy" OR "asthma" OR "chronic disease*")) AND (transfer OR transition)** Filters: **from 2023 - 2023**	267	04:39:43
#8	•••		Search: **((child OR children OR adolescent) AND ("diabetes type 1"[title/abstract:~2] OR "cerebral palsy" OR "asthma" OR "chronic disease*")) AND (transfer OR transition)**	4,697	04:39:27
#7	•••		Search: **(("Transition to Adult Care"[Mesh]) OR "Transitional Care"[Mesh]) AND ((("Chronic Disease"[Mesh]) OR "Diabetes Mellitus, Type 1"[Mesh]) OR "Cerebral Palsy"[Mesh])**	662	04:36:47
#6	•••		Search: **transfer OR transition**	1,315,827	04:35:36
#5	•••		Search: **("Transition to Adult Care"[Mesh]) OR "Transitional Care"[Mesh]** Sort by: **Most Recent**	3,337	04:34:54
#4	•••		Search: **"diabetes type 1"[title/abstract:~2] OR "cerebral palsy" OR "asthma" OR "chronic disease*"**	673,908	04:33:01
#3	•••		Search: **(("Chronic Disease"[Mesh]) OR "Diabetes Mellitus, Type 1"[Mesh]) OR "Cerebral Palsy"[Mesh]** Sort by: **Most Recent**	732,769	04:25:29
#2	•••		Search: **child OR children OR adolescent**	4,329,585	04:23:12
#1	•••		Search: **("Child"[Mesh]) OR "Adolescent"[Mesh]** Sort by: **Most Recent**	3,380,068	04:22:34

Showing 1 to 14 of 14 entries

FOLLOW NCBI

Connect with NLM

National Library of Medicine
8600 Rockville Pike
Bethesda, MD 20894

Web Policies
FOIA
HHS Vulnerability Disclosure

Help
Accessibility
Careers

NLM NIH HHS USA.gov
