# Supplementary material for: From pediatrics to adult care – Experiences of transition among youth with a chronic medical condition: A meta-ethnography
Source: Health Care Transit. 2025 Aug 27;3:100118. doi: 10.1016/j.hctj.2025.100118 (PMC12408251; doi:10.1016/j.hctj.2025.100118)
Supplement: Supplementary file 3 — Supplementary material [file mmc3.docx]

Suplementary File 1 Key concept from all the included studies

| **Key concepts from the index study**  **McLaughlin, 2013** |
| --- |
| Expert novices |
| Evidence and experience-based expectations |
| Negotiating new systems |
| Interdependence |
| Accepting less |

| **Key concepts**  **Strand et al., 2018** |
| --- |
| Taking responsibility for your own diabetes is a process |
| Taking responsibility for own diabetes is dependent on coping |
| It is demanding to take responsibility for own diabetes |

| **Key concepts**  **Lariviére-Bastien et al., 2013** |
| --- |
| Transition envisaged with fear and Apprehension |
| Lack of cooperation or communication between providers in the Pediatric and Adult healthcare systems |
| Lack of support, preparation, and information during the transition |
| Improper management and transfer og medical records |
| Difficulties related to the differences between the 2 healthcare systems |
| Abrupt loss of services, feeling a void at the time of transition |
| Feelings of abandonment during the transition |
| Sadness to leave the pediatric system |

| **Key concepts**  **Björnquist et al., 2014** |
| --- |
| Belonging to a family means security but may be “too much” |
| Socializing and experiencing love is necessary, but not always possible |
| Activities in daily life are manageable, but challenging |
| Surrounded by support, but what is going on? |
| Hopes for the future, but a desire for stepping-stone |

| **Key concepts**  **Leung et al., 2021** |
| --- |
| Individualization – how to personalize the transition experience |
| Identity – how the world relates to my diabetes |
| Interconnection – how my support system can help me with my diabetes |
| Impediment – how my diabetes limits me |

| **Key concepts**  **Iversen et al., 2019** |
| --- |
| Limited information about the transition |
| Transition from a frequent, thorough and personal follow-up to a less comprehensive and less personal follow-up |
| The importance of being seen as a whole person |
| Limited expectations of how the health services were organised |

| **Key concepts**  **Castensøw-Seidenfaden et al., 2016** |
| --- |
| Striving for safety |
| Striving for normality |
| Striving for independence |
| Worrying about future |

| **Key concepts**  **Olsson et al., 2023** |
| --- |
| **Dreaming of being nurtured towards self-reliance** |
| Struggling to find balance in daily life |
| Dealing with feelings of being different |
| Being supported to gradually achieve independence |
| Wishing to be appreciated as a unique person in healthcare |

| **Key concepts**  **Price et al., 2011** |
| --- |
| Living with asthma |
| Becoming adults with asthma |
| Asthma self-management |
| Parental involvement in asthma management |

| **Key concepts**  **Price et al., 2011** |
| --- |
| Appropriate adolescent health care |
| Recognizing individuality in health care |
