# Supplementary material for: From pediatrics to adult care – Experiences of transition among youth with a chronic medical condition: A meta-ethnography
Source: Health Care Transit. 2025 Aug 27;3:100118. doi: 10.1016/j.hctj.2025.100118 (PMC12408251; doi:10.1016/j.hctj.2025.100118)
Supplement: Supplementary file 4 — Supplementary material [file mmc4.docx]

| **1.Mclaughlin**  **2013**  **Index study**  **Score 9** | **2.Strand**  **2018**  **Score 8** | **3.Larivière-Bastien**  **2013**  **Score 7** | **4.Björquist**  **2014**  **Score 6** | **5.Leung**  **2020**  **Score 6** | **6.Iversen**  **2019**  **Score 6** | **7.Castensøe-Seidenfaden**  **2016**  **Score 5** | **8.Olsson**  **2023**  **Score 4** | **9.Rhee**  **2022**  **Score 4** | **10.Price**  **2011**  **Score 2** | **Towards third-order constructs*** | **Third-order constructs **** |
| --- | --- | --- | --- | --- | --- | --- | --- | --- | --- | --- | --- |
| **Ekspert novice**:  The young person with CP is an expert in their own condition and care, while also being a novice within the adult care system. Their lived experience with CP makes them experts, but the transition from paediatrics to adult care introduces uncertainty, thereby positioning them as novices once again |  |  |  | Identity: how does the world relate to my diabetes – Young people with diabetes experience unexpected and frustrating stigma and discrimination due to their diagnosis at both macro and micro levels, negatively affecting their quality of life. A negative social stereotype leads to exclusion and feelings of guilt, which they are left to manage and cope with on their own | Limited information about the transition – Young people with diabetes did not receive verbal or written information. They express a desire for repeated information, starting nine months prior to the transition |  |  | Self-administration – A paradox is highlighted between inadequate medical treatment and the young people's high confidence in self-administering their asthma care. |  | Development – Being in motion toward something new brings about a sense of dissolution |  |

| **Evidence and experience based expectations**: Young people with CP have experience-based expectations, learned in paediatrics, that their care goals include maintaining activity, mobility, and being pain-free, along with a continued need for support—but these goals are not met in adult care |  | Before transition – The transition is anticipated with anxiety: concerns centre on losing access to the professional support available in paediatrics. During transition – Young people struggle with the differences between the two systems (paediatrics and adult care), particularly the structural and relational discontinuities. Lack of time and resources in adult care – Paediatrics offers more personal attention and encouragement. After transition – There is an abrupt loss of services, accompanied by a sense of being avoided and rejected during the transfer period |  | Individualisation of transition options – There is no one-size-fits-all, as young people with diabetes prefer a multidimensional 'menu' of transition interventions, including optional support for coping strategies, communicating about their condition, workshops, or websites. Support should be tailored to the needs of the young person and their family |  |  | Young people with diabetes want a uniquely tailored approach from professionals but experience an abrupt transfer and standardised care that overlooks their individual needs; trust-based relationships with professionals are seen as essential. They struggle with feeling different, face ambivalence about disclosing their diagnosis, and seek strategies to come to terms with themselves | After transition – An abrupt loss of services is experienced, accompanied by feelings of being avoided and rejected during the transfer period | The transition process – From the perspective of young people with diabetes, the transition was perceived as an integrated part of their ongoing treatment trajectory. Experience and organisation of transfer – The way transfer is organised significantly influences each young person's experience. Information and education – Young people with diabetes are in a moratorium phase, requiring targeted support and information to navigate the transition | Transformation – is experienced as difficult. The difference between paediatric and adult care is challenging to navigate. There is a clear need for support in managing life with a diagnosis during youth. Cultural differences between paediatric and adult care reflect the healthcare system’s siloed structure, contrasting with the family-centred, patient-centred approach in paediatrics | **Navigating in the dark**  **Above all, being young** |
| --- | --- | --- | --- | --- | --- | --- | --- | --- | --- | --- | --- |

| **Negotiating new systems**:  Young people with CP experience transition as a process of 'negotiating new systems' represented by adult care, including insurance and healthcare—perceived in varied ways: as 'a bit over my head' or 'just another system to work within | Taking responsibility for one’s diabetes is a process – It feels natural for young people with diabetes to gradually take over responsibility for their care. They prefer early involvement to create a sense of security and prepare for the transfer, though the transition often results in a loss of the safety net when parents are no longer responsible.  Young people with diabetes seek greater independence and feel proud of managing their condition. Living with diabetes in adolescence is experienced as an unpredictable rollercoaster beyond the challenges of youth itself. Taking responsibility also implies personal blame when mistakes occur. | During transition – Lack of collaboration and communication between paediatric and adult care: there is insufficient cross-sectoral coordination between the two systems. During transition – Lack of preparation and information: young people report a general absence of information about the transition to adult care | Surrounded by support, but what is actually happening? – Inappropriate organisation: Young people reported a lack of awareness about available support services, which were also inaccessible due to being scheduled during school hours, making participation difficult | Barrier – how diabetes limits the young person: Diabetes is experienced as an irritating ‘extra burden,’ and the transition process is perceived as nerve-wracking and marked by a ‘loss of bond’ | Limited knowledge regarding healthcare system organisation: Young people accepted the organisational structure offered to them and held few expectations of their healthcare interactions |  |  |  | Organisation of professional services: Young people with diabetes perceive a difference in professionals’ approaches between paediatric and adult departments.  Inclusion: Some young people with diabetes require consultation support from family members, friends, or audio messages | Lack of continuity in the disease trajectory with ongoing development toward self-efficacy. Young people expect professionals to take responsibility. Transfer is more than just a specific age milestone | **Being the captain of ones’s own life**  **Above all, Being young** |
| --- | --- | --- | --- | --- | --- | --- | --- | --- | --- | --- | --- |

|  | Assuming responsibility is demanding – It requires knowledge, skills, and significant time investment. Managing diabetes independently is described as akin to being examined every single day |  |  |  |  |  |  |  |  |  | **Navigating in the dark** |
| --- | --- | --- | --- | --- | --- | --- | --- | --- | --- | --- | --- |
| **Interdependence**: Achieving autonomy is a process requiring preparation and influenced by parents, social norms, and external factors. Parents must be involved and not underestimated or excluded during transition – ‘we are all somebody’s kid.’ |  |  | Belonging to a family means security but can also feel like ‘too much’: family and staff (from Riksgym) provide crucial support.  Everyday activities are manageable yet challenging; young people with CP feel dependent. They can handle most tasks when planned and assisted but cannot tie their own shoes, cook, or make spontaneous plans to go to the cinema or pub | Connected with others: how relationships can help manage my diabetes: Young people with diabetes prefer mentors—either peers with diabetes or gradually supportive parents—as their connection to paediatric professionals shifts to adult healthcare providers, facilitating a successful transition |  | Striving for independence: Lack of knowledge, such as calorie counting skills, and insufficient professional support hinder young diabetics' autonomy.  Striving for security: Young diabetics perceive parents as a hotline providing a safety net, yet fear of complications often leads to inadequate actions. | A struggle to find balance in daily life. Diagnosis was life-changing, not always a straightforward process, with a hopeful yet challenging future.  Being supported to gradually achieve independence: early responsibility for self-management facilitates transition. Transition involves separation from family, yet young people experience parental emotional support as empowering support as empowering | Parental involvement in treatment: Despite young people’s developmental independence, they experience dependence on their parents in asthma management |  | Standing alone in uncharted waters |  |

| **Accepting less:**  The young people experience that transition culminates in lowered expectations of adult care due to disappointment in their encounters with the adult healthcare system |  | After transition, young people with CP experience an abrupt loss of services and feelings of being avoided and rejected during the transfer period, leading to ambivalence. They feel sadness and abandonment leaving pediatric care, which had felt like a supportive ‘family,’ making the transition, marked by the 18th birthday, emotionally difficult |  |  | Young diabetics experience an unexpected shift from frequent, thorough, and personalized professional follow-up in pediatrics to less understanding and impersonal care in adult services. They emphasize the importance of being seen as whole individuals, often feeling reduced to ‘just a number’ with impersonal interactions in adult care settings |  |  | Living with asthma involves emotional challenges and a significant responsibility for self-management. Young people transitioning to adulthood with asthma experience varying levels of concern about their diagnosis, and often communication with healthcare professionals is mediated through their parents, limiting direct interaction | Young diabetics desire a human, person-centered approach in professional consultations rather than a standardized communication model. They value individualized attention that acknowledges their unique experiences and needs beyond routine clinical procedures | Transfer is experienced as a gap or chasm between pediatric and adult care, where young people feel caught between two systems with different expectations and support functions. This creates uncertainty and a sense of loss during the transition | **Above all, Being young**  **Above all, Being young** |
| --- | --- | --- | --- | --- | --- | --- | --- | --- | --- | --- | --- |
| **Being a person with a disease** | Young people with diabetes desire greater independence and feel proud to manage their own condition. Diabetes during adolescence is experienced as an unpredictable rollercoaster, in addition to the challenges of being young. Taking responsibility also when you make a mistake |  | Socializing and experiencing love are necessary but not always possible: Despite being surrounded by caregivers, young people with cerebral palsy are uncertain about how to form friendships and romantic relationships |  | Longer and more guided nurse consultations were experienced as more satisfying and personal by the young people |  |  |  | The professional consultation: Young people with diabetes desire a human, person-centered approach rather than a standardized communication model | Being young, and secondarily living with a chronic illness |  |
| **Table 6 Matrice translationstable**  *Vertically, concepts related to the index study’s main concepts are presented, including the extraction of the concept Being a person with a disease*  *** *Horizontally refers to keywords representing progressive connections, towards third-order constructs*  *** Horizontally covers third-order constructs and entails cross-cutting categories* | | | | | | | | | | | |
